# Pattern of Deficient Mismatch Repair (dMMR)/ High Microsatellite Instability (MSI-H) Testing in India: A Questionnaire-Based Study

**DOI:** 10.7759/cureus.82627

**Published:** 2025-04-20

**Authors:** Amullya Pednekar, Sagar Bhagat, Saiprasad Patil, Nino Kurtsikidze, Hanmant Barkate

**Affiliations:** 1 Global Medical Affairs, Glenmark Pharmaceuticals Limited, Mumbai, IND; 2 Global Medial Affairs, Glenmark Pharmaceuticals Limited, Mumbai, IND; 3 Global Medical Affairs, Glenmark Specialty S.A., Basel, CHE

**Keywords:** dmmr, microsatellite instability, msi-h, msi testing, questionnaire

## Abstract

Introduction

Deficient mismatch repair (dMMR) and high microsatellite instability (MSI-H) constitute a unique phenotype within solid tumors, particularly colorectal, endometrial, and gastric cancer. They offer prognostic significance and serve as a predictor for responses to immunotherapy. The current study aimed to understand the practices, attitudes, and barriers associated with dMMR/MSI-H testing among medical oncologists in India.

Methods

This study was a cross-sectional survey utilizing a questionnaire, where Indian oncologists were selected through a convenience sampling method. A structured questionnaire consisting of 20 questions was developed, capturing comprehensive information on practices, preferences, and challenges faced by doctors during dMMR/MSI-H testing. A descriptive analysis was performed on the compiled data.

Results

A significant proportion of doctors (n=35, 62.5%) indicated a general preference for incorporating dMMR/MSI-H testing into their clinical practice. More than half of them believed that positive test results would be observed in 5-15% of patients with metastatic and non-metastatic colon and endometrial tumors. Immunohistochemistry (IHC) emerged as the preferred testing method, with 84% of oncologists usually conducting it as the first test. The issues of affordability and the subsequent awareness among oncologists emerged as a major barrier to the adoption of polymerase chain reaction (PCR) and next-generation sequencing (NGS) techniques for assessing MSI status. The vast majority (89%) agreed with the importance of detecting MSI status when assessing suitability for treatment with PD-1/PD-L1 inhibitors in solid tumor patients.

Conclusions

A deeper understanding of the importance of dMMR/MSI-H status in the clinical characteristics and prognosis of solid tumors, especially colorectal, gastroesophageal, and endometrial cancers, may lead to increased adoption of dMMR/MSI-H testing and guide the development of more effective therapies.

## Introduction

DNA mismatch repair (MMR) functions as a cellular mechanism post-replication that upholds DNA homeostasis and serves as a crucial element in ensuring genomic stability over evolutionary time. The primary role of the DNA mismatch repair system is to rectify spontaneous base-base mispairs and small insertion-deletion loops (indels) that predominantly arise during DNA replication. In instances of deficient MMR (dMMR), the system is unable to rectify these errors, leading to an increase in the cell mutational rate and alterations in the lengths of microsatellite sequences. Variability in microsatellite repeat lengths is termed microsatellite instability (MSI), a form of genomic instability that is a hallmark of tumor cells [1].

Three categories of MSI can be identified based on their frequency: high microsatellite instability (MSI-H), low microsatellite instability (MSI-L), and microsatellite stability (MSS) [2]. MSI-H predisposes tumor cells to the accumulation of somatic mutations and is associated with increased tumor mutational burden (TMB) and increased susceptibility to immune checkpoint inhibitors (ICI). MSI analysis plays a well-established role in detecting hereditary cancer syndromes (Lynch syndrome) and has prognostic importance in colorectal, small-bowel, endometrial, and gastric cancers. Furthermore, it shows potential as a predictive indicator of the response to immunotherapy [3-6]. Although ICIs have been accessible in India for the last five years, their prohibitive costs have posed a considerable obstacle to their extensive use [7], with one retrospective study highlighting immunotherapy access below 3% in India [8]. However, the anticipated increase in supportive government policies aimed at enhancing cancer care, coupled with the rising acceptance of cancer immunotherapy in India as well as the improved patient access programs from pharmaceutical companies, is projected to nearly double the immuno-oncology market within the country by 2030 [9].

Taking into account all the aforementioned factors, there has been a notable increase in the clinical demand for MSI molecular testing as a predictive biomarker for individuals who are likely to benefit from immunotherapy. Consequently, there is an immediate need to understand the prevalence of dMMR/MSI across various tumors in India, along with the testing methods utilized for its detection. This study was therefore undertaken to explore the knowledge, attitudes, practice patterns, and obstacles related to dMMR/MSI-H testing among medical oncologists in India.

## Materials and methods

This study was conducted as a cross-sectional, questionnaire-based survey aimed at understanding the current practices, preferences, and challenges associated with dMMR and MSI-H testing among medical oncologists across India. The study was conducted in April and May 2024 and involved oncologists with a minimum of 10 years of clinical experience. Participants were from private and academic/teaching institutions and represented all four geographic regions of India - North, South, East, and West - thus giving a broad and balanced representation of national testing practice.

A convenience sampling method was employed for participant recruitment. This non-probability sampling approach involved selecting a sample of oncologists from a readily accessible pool. While this method may not yield a fully representative sample, it enabled rapid data collection from experienced professionals with specific knowledge of dMMR/MSI-H testing. Given that the study did not involve any patient-related data, the methodology did not necessitate ethics committee approval.

A structured, self-administered questionnaire comprising 20 items was developed to systematically capture data across multiple domains. The survey instrument included questions on respondent demographics and professional background, indications and clinical rationale for ordering dMMR/MSI-H tests, testing methodologies and technologies utilized (e.g., immunohistochemistry, polymerase chain reaction (PCR)-based assays, next-generation sequencing), approaches to interpretation and clinical integration of test results, influence of testing on therapeutic decision-making, particularly in the context of immunotherapy eligibility, and institutional or logistical barriers faced in implementing widespread dMMR/MSI-H testing.

The survey used a combination of Likert scale scores, multiple-choice, and ranking questions to collect quantitative and qualitative data. To ensure the survey was relevant, understandable, and comprehensive, it went through a rigorous process of content validation. Five experienced senior medical oncologists reviewed the original version of the survey. Their feedback was used to revise and finalize the survey instrument to make it more scientifically and practically sound. 

The finalized questionnaire was disseminated electronically using SurveyMonkey®, a secure and widely used online survey platform. Access to the survey was restricted to invited participants through a unique survey link, and no personally identifiable information was collected. The distribution was executed via email and WhatsApp channels to a curated list of oncologists known to have experience or interest in dMMR/MSI-H testing. The survey design and execution adhered to the Checklist for Reporting Results of Internet E-Surveys (CHERRIES), ensuring high standards of transparency and reproducibility in web-based data collection.

All participants were provided with clear information regarding the objectives of the study, the voluntary nature of their participation, and how the data would be used. This information was presented at the beginning of the survey, along with a statement that the aggregated, anonymized data may be used for publication purposes. Informed consent was obtained electronically from each participant before they proceeded with the survey. Only those respondents who provided electronic consent were allowed to proceed to the questionnaire. To maintain data integrity and avoid duplicate entries, the survey link was restricted to one-time access per device. Follow-up reminders were sent via email at one-week intervals to optimize the response rate. The estimated time required to complete the survey was approximately 10 minutes.

**Figure 1:**
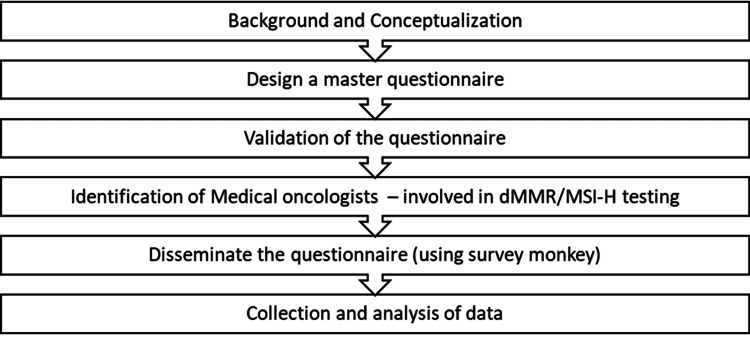
Methodology flow chart dMMR - deficient mismatch repair, MSI-H - high microsatellite instability

Upon closure of the survey window, responses were downloaded from the SurveyMonkey® platform and compiled into an Excel (Microsoft, Redmond, Washington) spreadsheet for further analysis. The dataset was cleaned, and responses were checked for completeness and consistency. Descriptive statistical analysis was performed to summarize the findings. Categorical variables were expressed as frequencies and percentages, and visual tools such as bar charts and pie charts were used where appropriate to enhance interpretability. All analyses were performed using Microsoft Excel (Figure 1).

Strict confidentiality was maintained throughout the data collection and analysis process. No personally identifiable information was collected, and all results are reported in aggregate to preserve respondent anonymity.

## Results

Out of the 60 medical oncologists who were invited to take part in the survey, 56 oncologists, accounting for 93% of the total, participated in the survey. Among the 56 doctors, 18 (32.1%) were from the West zone, followed by 16 (28.6%) from the North zone, 15 (26.8%) from the South zone, and seven (12.5%) from the East zone.

**Figure 2:**
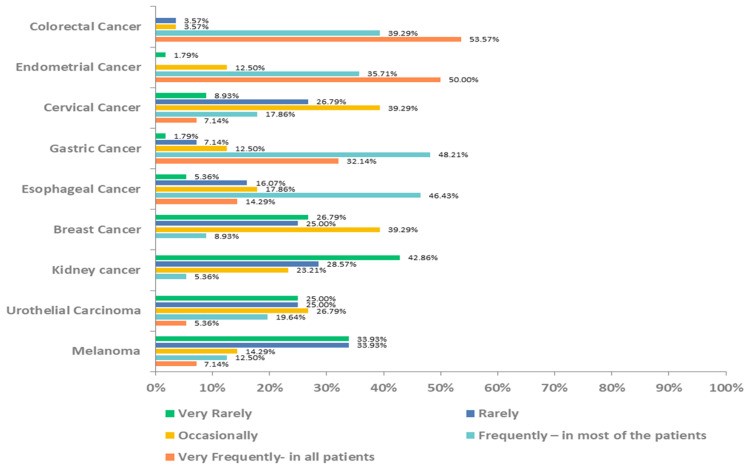
Frequency of dMMR/MSI-H testing in solid tumors dMMR - deficient mismatch repair, MSI-H - high microsatellite instability

The study findings indicated that a significant proportion (n=35, 62.50%) of these oncologists expressed a preference for dMMR/MSI-H testing in all patients diagnosed with solid tumors. A little over half of the oncologists responded that they carried out testing in almost all patients with colorectal and endometrial cancer, whereas a quarter of them were more inclined to do the same for cases of gastric cancer (Figure 2).

**Figure 3:**
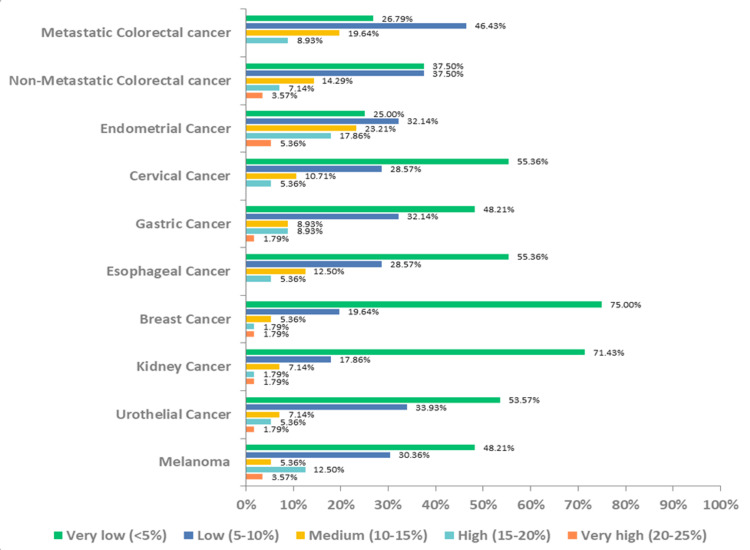
Incidence of dMMR/MSI-H positivity among solid tumor patients dMMR - deficient mismatch repair, MSI-H - high microsatellite instability

Nearly 66% (n=37), 52% (n=29), and 55% (n=31) of oncologists believed that positive test results would be observed in 5-15% of patients with metastatic colorectal cancer, non-metastatic colorectal cancer, and endometrial cancer, respectively (Figure 3). More than half of the doctors (n=33, 59%) initiated testing only after progression on first-line therapy (excluding immune checkpoint inhibitors), with the majority (n=43, 77%) considering it a standard procedure during tumor biopsy.

**Figure 4:**
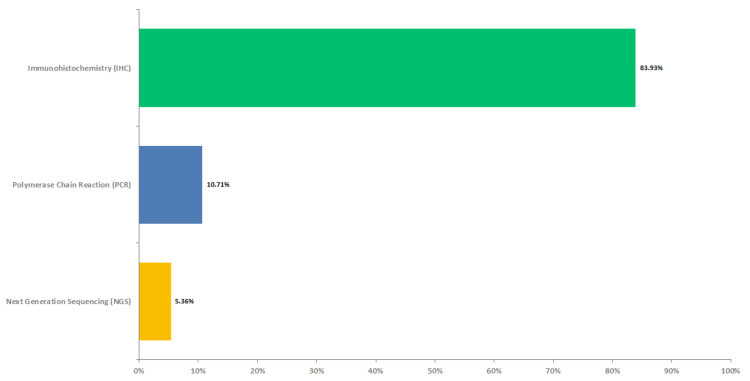
Initial test to confirm dMMR/MSI-H status dMMR - deficient mismatch repair, MSI-H - high microsatellite instability

Although 79% (n=44) of the doctors believed that next-generation sequencing (NGS) is a superior method with higher sensitivity than immunohistochemistry (IHC) and polymerase chain reaction (PCR) for testing dMMR/MSI-H status, IHC was the most popular testing method, with the majority of oncologists (n=47, 84%) typically performing it as an initial test (Figure 4).

**Figure 5:**
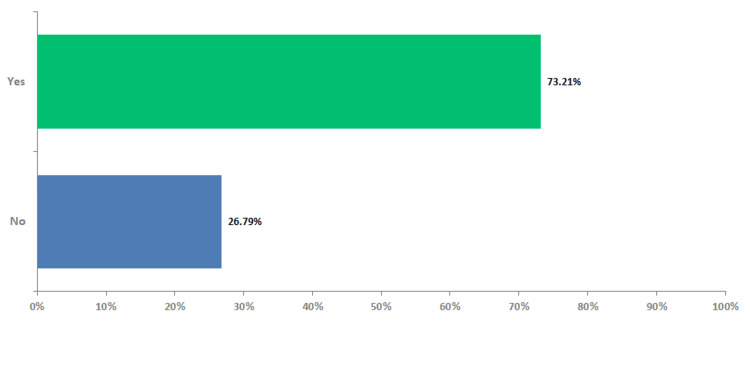
PCR exclusively in cases where dMMR IHC shows equivocal / questionable PCR - polymerase chain reaction, dMMR - deficient mismatch repair, MSI-H - high microsatellite instability

Most physicians (n=29, 52%) preferred primary as well as metastatic tissue samples for testing. IHC and PCR were performed together by very few oncologists (n=11, 20%) to confirm the diagnosis of dMMR/MSI-H status, with almost three-fourths preferring to exclusively perform PCR in cases where dMMR IHC shows equivocal/questionable results (Figure 5).

**Figure 6:**
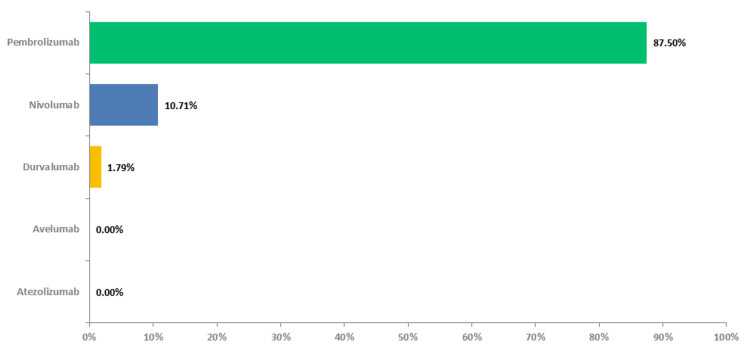
Preferred PD-1/PD-L1 inhibitor in dMMR/MSI-H patients dMMR - deficient mismatch repair, MSI-H - high microsatellite instability

The vast majority of oncologists (n=50, 89%) were in agreement that dMMR/MSI-H testing should be taken into consideration when assessing suitability for treatment with PD-1/PD-L1 inhibitors in patients with advanced solid tumors, with 88% (n=49) specifically endorsing the use of PD-1/PD-L1 inhibitors for this patient population, with pembrolizumab emerging as the preferred option (n=43, 88%) (Figure 6).

**Figure 7:**
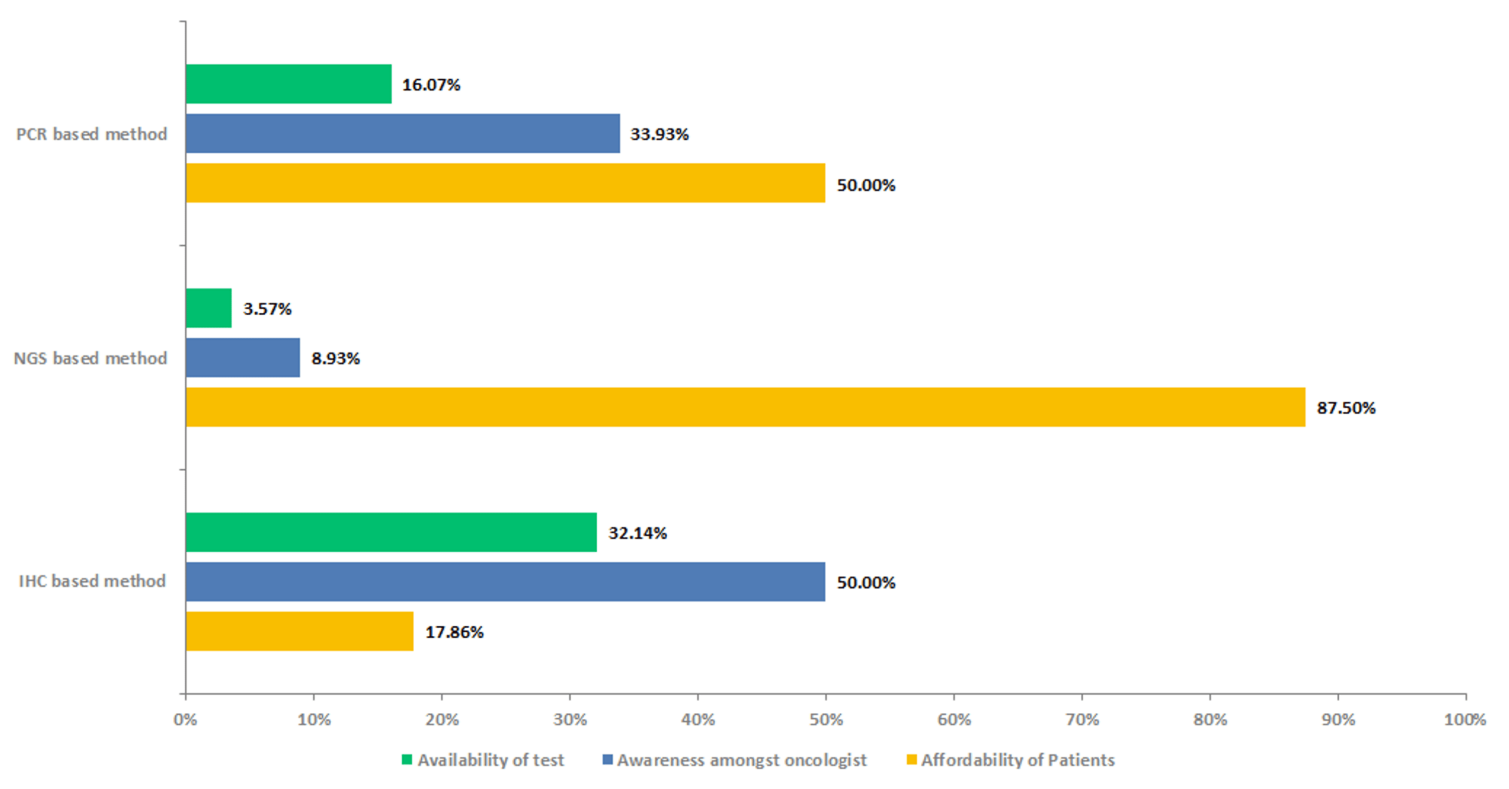
Need gap in the MSI-H/dMMR testing PCR - polymerase chain reaction, NGS - next-generation sequencing, IHC - immunohistochemistry, dMMR - deficient mismatch repair, MSI-H - high microsatellite instability

Awareness was a predominant reason for the gap in the adoption of IHC-based testing, while the issue of affordability came to light as a significant obstacle for PCR and NGS methods (Figure 7).

## Discussion

Among patients with solid tumors, particularly colorectal cancer (CRC), endometrial, and gastric cancer, dMMR/MSI-H status significantly improves overall survival rates when treated with PD-1 inhibitors, underscoring the importance of identifying dMMR/MSI-H status in solid tumor patients, given its presence across 25 different tumor types [10]. This survey-based research was a pioneering initiative aimed at examining the frequency of dMMR/MSI-H and diagnostic approaches utilized by oncologists in India for detecting dMMR/MSI-H.

The presence of MSI-H/dMMR in various solid tumors has led to a preference among most oncologists in this study to conduct MSI-H/dMMR testing in those patients, particularly those with colorectal and endometrial cancer. This aligns with the National Comprehensive Cancer Network (NCCN) guidelines, which recommend universal tumor MMR or MSI testing for all colorectal or endometrial cancers at diagnosis [11-12]. Moreover, the guidelines from the College of American Pathologists (CAP) endorsed by the American Society of Clinical Oncology (ASCO) recommend detecting DNA mismatch repair defects for those considered for ICI therapy [13]. Nevertheless, the current study found that oncologists in India preferred testing for MSI-H in all solid organ cancer patients, especially those in metastatic or advanced stages.

Our study emphasizes that IHC is the primary and most frequently conducted test for identifying dMMR status in patients, while PCR is reserved for cases where IHC results are inconclusive. On the other hand, NGS, which is known for its high positive and negative predictive values [14], is only performed when IHC results are inconclusive or contradict PCR. This approach aligns with ESMO's recommendation, which favors IHC for MMR proteins as the first test to assess MSI/dMMR, followed by PCR-based assessment of microsatellite alterations using five microsatellite markers, including at least BAT-25 and BAT-26 [15]. Furthermore, CAP, in conjunction with the Association for Molecular Pathology and Fight Colorectal Cancer, suggests that MMR-IHC and/or MSI by PCR are preferred over MSI by NGS for colorectal, gastroesophageal, and small bowel cancer. In the case of endometrial cancer, MMR-IHC is recommended over MSI by PCR or NGS for patients who are being evaluated for ICIs [16]. NGS, a relatively new test that combines MSI analysis with tumor mutational burden assessment [12], is considered more advanced than IHC/PCR tests. Still, the restricted application of NGS in India can be primarily linked to issues of affordability, as it tends to be more costly than IHC and PCR. This cost disparity is influenced by various factors, including the particular testing panel, the healthcare provider, and the geographical location within the country.

The timing of testing and the selection of optimal specimens are critical in the MSI-testing process [17]. The majority of oncologists opined that testing should be conducted when initial therapy (non-IO-based) shows progress, and it can be performed on samples from either the primary tumor or metastatic sites. Kawakami et al. [18] underscore the importance of early MSI/MMR testing in colorectal cancer patients, particularly when surgery or adjuvant chemotherapy is recommended. The incidence of dMMR/MSI-H is higher in the early stage (20%) compared to the metastatic stage (4%). The concordance of MMR/MSI status between the primary tumor and synchronous metastasis is not well understood [1]. In a study amongst 25 patients of mucinous adenocarcinoma of CRC, the concordance rate of MMR/MSI status between primary and metastases was 72.0% [19]. One study found matching IHC results between the primary tumor and metastatic tissue for all cases examined [20], while another study reported a 20.2% discordance in the MMR status between primary tumors and metastases [21].

Solid tumors with MSI-H exhibit dMMR, which leads to hypermutation and produces mutation-generated neoantigens that trigger an immune cell response. Therefore, patients with high MSI solid tumors are considered good candidates for immune checkpoint inhibitor (ICI) treatment [17]. Consequently, in 2017, the United States Food and Drug Administration (US FDA) granted approval for pembrolizumab and nivolumab as second-line treatment options for dMMR/MSI-H mCRC patients, with pembrolizumab later receiving first-line approval in June 2020. Keynote 164 and 158, using pembrolizumab, have demonstrated encouraging outcomes in both CRC and non-GE patients [22-23]. Similarly, Nivolumab has displayed improved survival rates in CRC patients (CheckMate 142 trial) [24]. It was observed in the study that the majority of oncologists, around 90%, opt for pembrolizumab when managing patients with MSI-H positive status. Amongst the landscape of ICI, the Chinese National Medical Products Administration (NMPA) granted marketing authorization to Envafolimab, the world's first single-domain PD-L1 antibody formulated for subcutaneous injection, as a second-line or further monotherapy treatment option for dMMR/MSI-H solid tumors, including advanced colorectal cancer, based on a pivotal phase 2 study [25,26]. Envafolimab could represent a significant advance in cancer treatment [25]. However, it still requires validation through more extensive, randomized controlled trials to establish its definitive role in cancer treatment. 

Approval of ICIs for dMMR/MSI-H advanced solid tumors highlights the significance of early testing for dMMR/MSI-H status to initiate therapy. However, challenges in the widespread use of these tests exists due to affordability as well as lack of awareness. In India, IHC is more commonly used due to its cost-effectiveness compared to PCR and NGS, but awareness remains a barrier to its widespread adoption. Enhanced comprehension of the significance of dMMR/MSI status in the clinical attributes and outlook of solid tumors, particularly CRC, gastroesophageal, and endometrial tumors, could potentially augment the utilization of dMMR/MSI testing.

Several limitations should be noted in this study. The convenience sampling method used may introduce selection bias, as the sample may not fully represent all oncologists in India. The study focused on knowledge, attitudes, and practices regarding dMMR/MSI-H testing but did not assess clinical outcomes resulting from these testing practices. Our study pinpointed cost and knowledge as major obstacles to the extensive implementation of cutting-edge testing techniques such as NGS. However, it did not explore in-depth the socioeconomic factors that might influence these barriers across different regions and healthcare settings in India. The study acknowledged the inclination towards IHC because of its cost-efficiency. Yet, it does not examine the technical constraints and variations in the effectiveness of different testing methods (IHC, PCR, NGS) in various laboratory settings. The emerging role of liquid biopsies, particularly the use of circulating free DNA (cfDNA) for dMMR/MSI-H testing, was out of scope in our study. Moreover, liquid biopsies were not included in this study due to limited infrastructure, high costs, and lack of standardized protocols in the Indian setting. Liquid biopsies offer a non-invasive alternative to tissue biopsies, identifying patients whose tumors harbor biomarkers of interest not otherwise identifiable due to tissue sampling limitations, and doing so more rapidly. Liquid biopsies thus have the potential to expand patient access to standard-of-care-targeted therapies, including ICIs. This study did not assess how the choice of dMMR/MSI testing methods impacts clinical outcomes, which limits our ability to correlate diagnostic practices with patient management or treatment efficacy.

Future studies should address the limitations of this research by employing stratified sampling, which could enhance the representativeness of the data by ensuring proportional inclusion of oncologists across various regions and healthcare settings. This approach will help control for geographic and institutional variations in MSI testing practices, leading to more generalizable insights. Additionally, a deeper exploration of socioeconomic barriers, technical performance variations of testing methods (IHC, PCR, NGS), and the emerging role of liquid biopsies using cfDNA is necessary to enhance patient access to advanced therapies and improve testing practices nationwide. Moreover, future studies are warranted to explore the association between testing practices and clinical outcomes, which would provide deeper insights into the real-world impact of dMMR/MSI testing on patient care. 

## Conclusions

Our study highlights that most oncologists in India prefer to perform dMMR/MSI-H testing in patients diagnosed with colorectal cancer and endometrial cancer. These tests are typically carried out after initial therapy shows progression, either from the primary tumor or a biopsy of a metastatic lesion. Immunohistochemistry (IHC) is the preferred test among oncologists. Still, its broader use in MSI-H diagnostics is currently limited by a lack of awareness amongst peers, patient affordability, and accessibility.
